# Patient lumbar discectomy journey (DiscJourn) in the UK: a qualitative study

**DOI:** 10.1136/bmjopen-2025-101259

**Published:** 2025-07-25

**Authors:** Louise White, Nicola R Heneghan, Navin Furtado, Karl Baraks, Zeeshan Parvez, Annabel Masson, Alison B Rushton

**Affiliations:** 1 Physiotherapy Department, Level 1, North Suite, Queen Elizabeth Hospital, Birmingham, UK; 2 Queen Elizabeth Neurosciences Centre, Queen Elizabeth Hospital, Birmingham, UK; 3 Centre of Precision Rehabilitation for Spinal Pain, School of Sport Exercise and Rehabilitation, University of Birmingham, Birmingham, UK; 4 Department of Physiotherapy, Sandwell and West Birmingham Hospitals NHS Trust, Birmingham, UK; 5 Department of Allied Health, Birmingham Newman University, Birmingham, UK; 6 School of Physical Therapy, Western University Faculty of Health Sciences, London, Ontario, Canada

**Keywords:** Spine, QUALITATIVE RESEARCH, Patient-Centered Care

## Abstract

**Abstract:**

**Objectives:**

To gain insight into patients’ views, perceptions, experiences and expectations postlumbar discectomy.

**Methods:**

A qualitative study using interpretative phenomenological analysis (IPA) purposively recruited patients undergoing lumbar discectomy at one UK spinal centre. Purposive criteria included age, sex, ethnicity, symptom duration, work/sick leave, education level and co-existing psychological issues. Semi-structured interviews were conducted using a patient co-constructed topic guide. Interview transcriptions were analysed in accordance with IPA. Strategies enhancing trustworthiness included suspension of judgements and presuppositions, reflexivity, iterative coding process and critique from co-investigators.

**Results:**

Data from 14 participants (eight elective, 6 emergency surgery) informed four themes. The theme ready to move forwards was characterised by high satisfaction with post-operative improvement, positivity and optimism, with readiness to work towards personal goals. The theme post-operative fear and uncertainty was characterised by reflections on pre-operative difficulties fuelling fear about potential recurrence and long-term impacts. The theme of advice and guidance considered important was characterised by the expectation and value of support provided (verbal, written); instances of negative influences from healthcare interactions and access to unregulated patient information sources suggest scope for future improvement. The final theme, heterogeneity in peri-operative needs, was characterised by variation in depth/access to patient information, perceived post-operative support and wide-ranging preoperative activity/fitness.

**Conclusions:**

Surgery offers physical and psychosocial changes which could be better harnessed to positively influence recovery through high quality verbal/written communication. Peri-operative advice and guidance was valued; while this was sufficient for some, personalised rehabilitation should be available owing to the identified heterogeneity.

**STRENGTHS AND LIMITATIONS OF THIS STUDY**

- The use of interpretative phenomenological analysis (IPA) was a key strength with this methodology enabling exploration of variation in individual experiences during the transition from prediscectomy surgery through early postoperative recovery.
- The main author was immersed in the study through data collection and subsequent analysis, although analysis was collaborative, including researchers, clinicians and patient representatives, with this triangulation strength.
- Efforts were made to include a representative sample of discectomy patients including those undergoing emergency and elective procedures with wide-ranging backgrounds.
- A limitation was that none of our participants described dissatisfaction or specifically experienced perioperative complications.
- All participants underwent surgery within one secondary care NHS Trust, although external validity is a recognised limitation of IPA.

## Background

 Lumbar discectomy is an internationally recognised surgical procedure for discogenic neural compression causing concordant severe or progressive neurological deficits, especially when conservative treatments have been exhausted.^1^ Discectomy is the most frequent spinal surgical procedure^2^ with moderate-level evidence of postdiscectomy improved leg pain and disability as well as associated reduced back pain.^3^ Furthermore, analysis of world-wide patient-rated outcomes revealed 79% excellent-to-good results postdiscectomy,^2^ consistent with earlier studies where 69%–79% reported ‘good recovery’ 1 year postdiscectomy^4^ and 78%–95% success at 1–2 years post-op.^5^ Despite these favourable results, not all patients experience full symptom resolution and functional recovery. A recent study reported 26.3% and 30.6% of participants describing poor improvement in leg and back pain, respectively, postdiscectomy,^6^ with persistent mild-to-moderate disability after 2–8 years^3^ and 5 years.^7^ A systematic review^8^ revealed widely varying work rates (3%–100%) within 24 months of surgery for radiculopathy and sick leave variation of 0.8–20 weeks. Collectively, findings illustrate significant variation in recovery, which is currently not well understood.

Evidence guiding perioperative management is limited. Systematic reviews identify low/very low-quality evidence demonstrating potential to improve pain and disability,^5^ movement and physical impairment^9^ in the short-term with high-intensity rehabilitation exercise programmes commencing 4–6 weeks postsurgery. Also, there is ongoing debate about support that clinicians think patients need and postsurgery activity restrictions.^10^ Additionally, reports suggest poor quality discectomy patient information with lack of perioperative information contributing to depression and anxiety both before and after surgery^11^ and such emotions associated with poor surgical results. This is concordant with the wider evidence base, with reports of negative emotions increasing the risk of chronicity and disability^12^ and psychological processes potentially catalysing the transition from acute to chronic pain. Therefore, exploring the patients’ perspective, in terms of perioperative rehabilitation needs (including exercise, advice, guidance and information expectations) as well as the effect of perioperative support on recovery, requires deeper understanding.

Within the existing evidence base, qualitative studies are rare yet offer in-depth understanding of patients’ experiences and have been associated with the development of high quality, safe and clinically effective care.^13^ One interview study explored acceptability of outpatient discectomy surgery, finding positive patient experiences including satisfaction with level and quality of perioperative information, but rehabilitation experiences were not included.^14^ Another semistructured interview study^15^ reported postoperative movement restrictions due to participants’ high anxiety and fear of re-injury with lack of physiotherapy support to explore optimal activity and functional recovery. However, this study included eight elective patients excluding^15^ those undergoing emergency surgery, with postoperative focus on physiotherapy rather than considering wider influences on patients’ beliefs and behaviours. Also, a focus group study found differences in management preferences within and between patient and physiotherapist groups,with some patients indicating that ‘advice and guidance only’ was acceptable for them.^16^ Previous studies^5 9^ have assumed that all patients need and want intensive postdiscectomy rehabilitation, but there may be some heterogeneity within perioperative needs. Building on existing evidence, the aim of this study was to understand patients’ perspectives and lived experiences of lumbar discectomy surgery.

### Objectives 

To explore the patient discectomy journey and understand experiences relating to symptomatology and function.

To capture the patients’ perceptions of the need for, value of and adherence to perioperative care and guidance.

To understand the patient journey through return to functional activities and gain insight into barriers and facilitators affecting recovery, similarities and differences between patients/ patient groups (eg, elective vs emergency) and perceptions relating to post-op rehabilitation and recovery.

## Methods

### Design

A qualitative study employing an Interpretive Phenomenological Analysis (IPA) approach enabled rich exploration of the patient’s experience (phenomenology) combined with making sense of the experience (hermeneutics).^17^ Study design and data analyses were conducted in partnership with two patient co-investigators according to a published protocol^18^ reported in line with Standards for Reporting Qualitative Research^19^ and Consolidated Criteria for Reporting Qualitative Studies.^20^ A Study Management Group (SMG) including clinical physiotherapists (LW, KB), academic researchers (ABR, NRH), Patient and public involvement (PPI) representative and post-graduate physiotherapy student (ZP) met regularly to oversee study quality.

### Participants

A purposive sample of 14 patients (≥16 years) undergoing primary lumbar discectomy surgery (emergency or elective) and able to communicate in English was recruited in one UK secondary care spinal surgery setting. Sampling ensured a range of participant characteristics, such as age, sex, ethnicity, symptom duration, work satisfaction, sick leave duration from work, educational level, co-existing psychological issues and coping strategies. Exclusion criteria included infection, malignancy or previous surgery. Potentially eligible participants were identified (n=22) by neurosurgery team members including hospital Principal Investigator (PI) (LW), surgeons, ward physiotherapists and waiting list coordinators prior to or during admission.

### Data collection

Semistructured interviews were arranged and undertaken by the PI (LW), a specialist physiotherapist, between May 2018 and March 2019. Participants were aware of the interviewer’s background with interviews completed at participants’ home or hospital within 3 weeks of surgery. The topic guide (box 1 and online supplemental file 1) was co-constructed drawing on evidence and insights from patient co-investigators. Pilot interviews (n=2) were undertaken to ensure quality of the topic guide and practice, with no changes made. Interviews were audio-recorded and professionally transcribed verbatim.

Box 1 **Topic Guide sections**

**Topic guide sections**

Pre- and postoperative experiences

Participant’s expectations from surgery

Underlying attitudes and beliefs towards the surgical intervention

Facilitators and barriers to recovery

Adherence to advice and physiotherapy; experiences of rehabilitation

Return to previous function, activity and/ or work

### Data storage and management

Adherence to the Research Governance and Data Protection Act 2018 included electronic records storage on password-protected computers, preserved and accessible to the research team for 10 years poststudy completion. To maintain participant pseudoanonymity, numbers were used to depersonalise data. Transcribed texts were checked with audio recordings and file notes prior to analysis.

### Data analysis

Analysis was undertaken in accordance with IPA.

*Stage 1*: Interviews transcribed verbatim were combined with interviewer’s observations, reflections and notes.

*Stage 2*: In accordance with IPA, two investigators (LW and ZP) initially independently immersed themselves in data reading and re-reading transcriptions combined with listening to recordings. Noting included descriptive, linguistic and conceptual comments which were then organised to develop emergent themes.

*Stages 3 and 4*: The PI (LW) and blind reviewer (ZP) independently grouped emergent themes as clusters and tabulated them in a summary table, illustrated by verbatim extracts. Themes were then presented and discussed with the SMG and checked for consistency and accuracy. The SMG provided critique with discussion to consider a priori concepts throughout the process.

### Patient and public involvement

This project involved two patient and public representatives from inception. Both PPI representatives had undergone lumbar discectomy surgery and one contributed to previous lumbar discectomy projects undertaken by the research group. As co-investigators, patient representatives contributed to the interview topic guide, participant information sheets and consent forms. They were integral members of the SMG and therefore in data analysis.

### Trustworthiness

Strategies to ensure trustworthiness^17^ included researchers discussing their preconceived beliefs, knowledge and clinical experience openly and acknowledging potential impact on data. Other strategies included rigorously checking/rechecking data to the level of minor themes with blind coding from two researchers (LW, ZP), regular discussions with SMG and code–recode audits. Coding was an iterative process including collaborative analysis with triangulation of analysis and interpretation through regular discussions with fellow researchers, enabling greater transparency of data discussed within the SMG. This ensured peer and patient critique and review, thus providing professional and PPI perspectives enhancing researcher reflexivity and quality of analysis.

### Ethical considerations

Informed consent was sought during admission for surgery. Recruiters had current Good Clinical Practice (GCP) training. Ethical approval was granted by the London-Bloomsbury Research Ethics Committee (18/LO/0459; IRAS 241345). Hospital Research and Development approval was granted.

## Findings

### Participants

Twenty-two patients were introduced, with six declining participation. Two were excluded as they underwent more extensive surgery. Participants (n=14) included n=8 females, aged 26–76 years (median 51 years). Eight underwent elective surgery (Elsurg) and six emergency surgery (Emsurg) (table 1). Further details of participants are available in online supplemental file 2.

**Table 1:** Participant Demographics

		N (%)
Age (years)	20–35	4 (28.6)
	36–50	6 (42.8)
	50–70	3 (21.4)
	>70	1 (7.1)
Sex	Female	6 (42.8)
	Male	8 (57.2)
Ethnicity	White British	12 (85.7)
	British Asian	2 (14.2)
Level of surgery	L4/5	6 (42.8)
	L5/S1	8 (57.2)
Elective or emergency	Elective	8 (57.2)
	Emergency	6 (42.8)
Symptom duration	0–1 year	6 (42.8)
	1–2 years	5 (35.7)
	>2 years	3 (21.4)
Employment status	Employed/self-employed	10 (71.4)
	Unemployed	2 (14.2)
	Retired	2 (14.2)
Co-existing past medical history	Depression	2 (14.2)
	Panic attacks	1 (7.1)
	Cardiac	1 (7.1)
	Nil	10 (71.4)

### Themes

**Figure 1:**
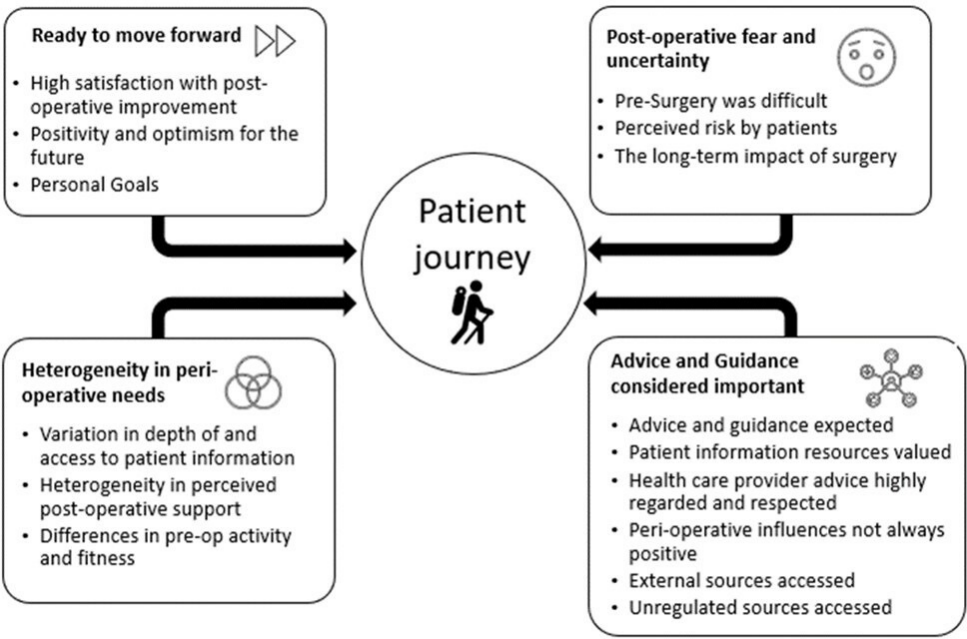
Themes and subthemes.

Four key themes emerged from participants’ experiences during early post-op recovery: ready to move forward, postoperative fear and uncertainty, advice and guidance considered important and heterogeneity in perioperative needs (figure 1).

#### Ready to move forward theme

Participants described confidence that surgery had been necessary and the right decision. Physical and psychological improvements were identified and, with surgery behind them, participants were motivated to engage with recovery and ready to move forward with their lives. Three subthemes characterised this theme (table 2).

**Table 2:** Subthemes for ready to move forward

Subtheme	Description	Illustrative quotations
High satisfaction with postoperative improvement	Participants described reduced leg pain, numbness, paraesthesia and/or weakness following surgery. Six participants described that expectations were surpassed with instances of surprise at the immediate improvements experienced.	P1 (Elsurg) *“I suppose straightaway noticing I had got no pins and needles in my leg…it was fantastic, amazing.”* P9 (Emsurg) *“I'm pain free, that’s the main thing, I'm happy. And that’s why I'm positive”* P4 (Elsurg) *“My expectations post-surgery were well exceeded.”* P13 (Elsurg) *“I didn’t think it would be as good as what it is.”* P16 (Emsurg) *“I was quite surprised at how mobile I was…. Pain-wise really, I was quite surprised at how little pain I was in.”*
Positivity and optimism for the future	Participants reported psychological positivity and optimism for the future; they could see hope to return to “normal life” including activities valued and enjoyed. There was perceived opportunity to improve quality of life with associated motivation and enthusiasm for the future. There was only one exception; one Emsurg participant described post-operative shock and negative emotions related to sudden progression resulting in surgery. The very limited time to prepare contributed to shock with realisation about the impact to normal activities.	P4 (Elsurg) *“There were no negative feelings… all on the plus side…”* P5 (Emsurg) *“I think generally I’m all positive… because the achievement that we’ve had, having this procedure, gives me a light at the end of the tunnel, which I haven’t had. This is like seriously moving forward for me…. I’m feeling really positive.”* P10 (Elsurg) *“I'm a hell of a lot better than I was before the operation, already….but yeah, I hope to go back to work, go back to socialising. I might even join a gym….”* P12 (Emsurg) *“I felt quite down a few days after…… I’ve gone from being the nurse to being the patient … it was a shock for me to be able to come to terms with…. It was just the realisation that I’m human. You know, I’ve just got to take this on board that it’s going to take a bit of recovery.”*
Personal goals	Participants all described how their lives had been restricted because of their symptoms. Returning to meaningful and enjoyable activities was very important and fuelled their drive to achieve personal goals.	P5 (Emsurg) *“I have to go back, I will rush the process because I need to get back to work, I need to get back to that.”* P8 (Elsurg) *“My goal is … I want to be 75% back to where I was before and if I can get at least 75% of where I was with my running, going to the gym, my walking, I’ll be happy. Anything above that will be a bonus.”* P3 (Emsurg) *“I just needed it (surgery) done so that I could get back to work.”*

#### Post-operative fear and uncertainty theme

Participants described fear and uncertainty experienced concurrently with postoperative positivity. Three subthemes characterise this theme. The difficulties associated with preoperative life fuelled dread of recurrence or relapse with acknowledgement of the holistic negative impact and approximately half the participants reflected that they were not coping presurgery. Subthemes also characterise the implications for how associated beliefs influenced immediate and longer-term future. Furthermore, reflection on presurgical difficulties underpinned concerns that participants could cause setbacks or recurrencies if the ‘right rules’ and restrictions were not followed as well as thoughts and beliefs affecting future behaviours (table 3).

**Table 3:** Subthemes for fear and uncertainty

Subtheme	Description	Illustrative quotations
Pre-surgery was difficult	Participants reflected on presurgery difficulties: Symptoms were escalating. Loss of function. Impact on others and loss of independence. Negative psychological effects (including anxiety, depression, frustration). Not coping.	P8 (Elsurg) *“I was in that much pain, it was unbelievable… more leg (pain)… I started to get ….foot drop. I couldn’t control my foot.”* P6 (Elsurg) *“No sleep… you’re going on a family day out, I can’t go. I couldn’t do anything like that…”* P16 (Emsurg) *“I must have been the worst person to live with … I’m not used to being reliant on people and I literally became reliant on everybody to do everything…it’s been a tough time, really tough.”* P7 (Elsurg) *“when the anxiety comes in….that’s the main reason why I wanted to just stop in the house.”* P4 (Elsurg) *“It really made me feel miserable. Really unhappy, depressed.”* P6 (Elsurg) *“Do I have to throw myself in front of an ambulance on its way into A&E to get myself seen? That’s what it felt like.”* P3 (Emsurg) *“I’d just completely had enough. I’d given up…. It’s the only thing left I could do…”* P13 (Elsurg) *“I couldn’t take it any more. I’d had enough.”*
Perceived risk by patients	Participants voiced fear and uncertainty that their actions could cause regression thus decreasing confidence to self-manage. There were vulnerabilities and concerns that improvements would not be permanent, especially if they did not follow the right guidance. There were also instances of participants feeling they could do more but limiting activities based on perceptions that this would reduce the likelihood of developing setbacks or recurrence.	P16 (Emsurg) *“I don’t want to jeopardise anything…. I was just conscious that I didn’t want to aggravate anything. I was probably being over-cautious to be honest….this has got to be right.”* P8 (Elsurg) *“I’m scared to push myself …. You read about these things in booklets saying Oh I’ve started too soon and I’ve ended up having another operation.”* P4 (Elsurg) *“I’m concerned that I may do more harm than good if I try and push it too quickly…its miserable sitting here twiddling my thumbs…”* P7 (Elsurg) *“I’m scared to push myself …. I think, well, I’m going to pace myself.”*
The long-term impact of surgery	Participants described their beliefs and concerns about the impact of surgery and the associated influence on future behaviours aiming to mitigate recurrence or regression.	P15 (Emsurg) *“I don’t really know what’s going to happen like further down the line, I’m not sure.”* P3 (Emsurg) *“Because he’s taken out of my disc, it will never be 100% now. It will always be a worry of it going again…. So I’m always going to be very wary of what I do.”* P12 (Emsurg) *“Its how its all going to pan out for the future which is a bit scary really….”*

#### Advice and Guidance considered important theme

This theme was characterised by participants’ descriptions of perioperative advice and guidance expectations and experiences to navigate recovery and optimise outcomes. Overall advice and guidance were considered important with six subthemes characterising this theme (table 4).

**Table 4:** Subthemes for Advice and Guidance important

Subtheme	Description	Illustrative quotations
Advice and guidance expected	Participants looked to the Healthcare Team (HCT) to provide the right advice and guidance to support recovery. Participants described strong compliance with guidance provided.	P6 (Elsurg) *“When can I start moving to bend? How fast paced, how much do I walk? How much am I allowed to walk?”* P9 (Emsurg) *“…any kind of information or guidance they've given me at the hospital I followed it.”* P13 (Elsurg) *“Exactly doing whatever they tell me to do.”*
Patient information resources valued	Participants described patient information resources as useful to reinforce the verbal advice provided in face-to-face consultations.	P12 (Emsurg) *“I got given the leaflet which I went through, which was useful because obviously you don’t digest it all.”* P16 (Emsurg) *“(surgeon) gave me a website to look at which detailed the operation, risks and stuff like that.”* P4 (Elsurg) *“(Surgeon) gave me a couple of websites to look at…to research it more….It’s a time-bound thing…he’s got to get on and do surgery….”*
Healthcare provider advice highly regarded and respected	When unexpected symptom increases were experienced, anxiety of triggering a self-induced setback led to seeking advice and guidance. Follow-up with the surgical team was valued for peri-operative guidance and reassurance that progress was as expected. For some, this contact was considered necessary for approval to increase activities.	P6 (Elsurg) *“I’d done a bit too much… crouched down… felt a twinge, then I thought oh god….and then had pain shooting down my leg. I phoned (surgeons secretary) I was like, I hope I haven’t done it.”* P8 (Elsurg) *“it’s good to talk to someone who knows what you can do and can’t do.”* P7 (Elsurg) *“I think it might change when I actually see that surgeon…. to say that Yeah, everything went great… I think it will give me more motivation to start doing things again.”*
Peri-operative influences not always positive	Participants also described inadvertent advice potentially amplifying negative beliefs and behaviours (eg, restricting and avoidant behaviours).	P4 (Elsurg) *“I’m taking medical advice and (the physiotherapist) said…. You could drive after 2 weeks but I don’t recommend that you do for at least six. So that’s what I’m doing.”* P2 (Elsurg) *“When I went and had my staples out… she (nurse) said I’ve got to be very careful because there’s nothing holding the wound together now, which has made me a little bit on edge…. so I’m scared of pulling my own wound apart.”* P3 (Emsurg) *“That’s when the female said, “I can’t do anything yet”……She said “You shouldn’t be doing this. It’s too soon””*
External sources accessed	When information and guidance was insufficient to meet needs, external sources were sought (including websites and chat forums). Some described critical analysis of resources, for example cross-referencing prior to emergency surgery for deteriorating lower limb weakness; another undertook detailed research recognising that resources did not always align to local UK guidance. However, methodical searching and critical analysis of sources was not undertaken by all.	P15 (Emsurg) *“I’d googled what a discectomy was…“* P6 (Elsurg) *“Oh there is nothing on the internet I haven’t read about…. I’d watched the operation, I watched a live operation on YouTube, read everyone’s (posts) on YouTube.”* P15 (Emsurg) *“I’d looked at a lot of different ones to make sure I wasn’t getting… a biased view”* P3 (Emsurg) *“A lot of it was from America. Over there I think it was quite different….they were out for like 3 months for rest.”* P4 (Elsurg) *“There are websites a plenty. A lot of them American….They do tend to be contradictory. I do wish people would start talking to each other…. This is what the Royal College of surgeons say, this is what the people in America say. This is what the people in Germany say. You’ve got to try and make an informed decision of all this information that you’re getting…”* P6 (Elsurg) *“At least I can log onto Google … John in Connecticut has had a bit of shooting pain in the back of his leg and don’t worry about it. His doctor said it was the nerve repairing.”*

#### Heterogeneity in perioperative needs theme

Although all participants underwent the same surgical procedure, heterogeneity evolved as a theme in relation to participants’ perioperative needs characterised by three subthemes (table 5).

**Table 5:** Subthemes for heterogeneity in perioperative needs

Subtheme	Description	Illustrative quotations
Variation in depth of and access to patient information	Although peri-operative advice and guidance was valued by all there was significant variation in preferred depth and type of delivery of guidance. Some looked for very detailed guidance. Conversely, two participants described relying *only* on verbal advice and guidance provided within clinic consultations. There were indications of consequent limited understanding of peri-operative expectations including post-op milestones and indications that this approach adversely influenced self-management. There was also variation between patient information provision for Elsurg and Emsurg participants; emergency surgery patients described “missing out” on preparation and subsequently seeking more post-operative information and guidance.	P12 (Emsurg) *“I got given the leaflet which I went through, which was useful because obviously you don’t digest it all.”* P4 (Elsurg) *“My wife and I looked at various aspects of it (patient information)… there are websites aplenty…”* P2 (Elsurg) *“I was happy with what he (surgeon) spoke to me about and I was happy to go with that.”* P2 (Elsurg) *“I don’t know…. I’m still washing up. Whether I am supposed to do that or not, I don’t know…… I didn’t think I’d be walking and I didn’t think I’d be as mobile as well as I am. I thought I’d be bedbound…. you do like a quick walk and you go back to bed. That’s what I thought…… I’m sure they said for full recovery its three months. But I don’t know what in between is… what I’m allowed to do. That wasn’t discussed…. but from six weeks to the three months what am I allowed to do in between that space?”* P12 (Emsurg) *“I guess being an emergency I missed out on that phase (pre-op preparation) so now it just feels like a long time (for post-op review).”* P9 (Emsurg) *“I asked a lot of questions …. it was happening very, very fast, in my mind I had to know the answers…… I didn't know what to expect. It was just like it happened very quick.”*
Heterogeneity in perceived post-operative support	Some participants described satisfaction with only post-operative advice and guidance. Conversely, others expected more intensive rehabilitation with detailed structure. Others looked for support to guide rehabilitation at an appropriate pace and also motivation and encouragement to progress. There were accounts of transferable skills improving confidence to self- manage, for example, from pre-surgery physiotherapy and exercise (P1), transfer of Cognitive Behavioural Therapy knowledge (P4) for previous depression treatment and training/ life-long exercising (P8).	P3 (Emsurg) *“They gave me a booklet of stuff I needed to do, what I shouldn’t do. Just follow that.”* P1 (Elsurg) *“I’d probably be alright…. It’s just for a bit of reassurance.”* P4 (Elsurg) *“I think I need advice on what leg exercises …Back strengthening exercises to do*. *Frequency and repetitions… I want to be able to go through a measured period of rehabilitation so that I can get my physical strength back.…”* P16 (Emsurg) *“(The physiotherapist) will push me, put me through my paces when she thinks I’m ready.”* P1 (Elsurg) *“I’m doing the walking, and I’ve started adding in little exercises, just ones done previously, clam leg things… some basic core stability things, basic Pilates things.”* P4 (Elsurg) *“… The coping strategies that I’d learned from my talking therapy for stuff that I’ve dealt with myself over the years.”* P8 (Elsurg) *“That comes from going to the gym 20–30 years. It’s that knowledge where other people who maybe don’t go to the gym, I think it might be a lot harder because they don’t know what their limitations are and where to stop.”*
Differences in pre-op activity and fitness	Participants described wide-ranging pre-surgery activity levels suggesting inevitable differences in fitness. Illustrating the “active pre-op” end of the spectrum, two Emsurg participants experienced sudden deterioration with no major pre-existing symptoms with three Emsurg participants at work on day of admission (P5, P12 and P15). Conversely, 2 others (P2, P3) described persistent sedentary pre-operative lifestyles.	P15 (Emsurg) *“I’d worked quite a lot in the week… Saturday, Sunday, Monday (day attended ED) were the worst three days. I could manage up till that point.”* P4 (Elsurg) *“I tried to make myself as fit as I possibly could.”* P2 (Elsurg) *“I was sleeping in the living room on the mattress…. I’d have to lie on my front and ate off the floor… because I was in that much pain…. I was lying down quite a lot through the day … or on my side.”* P3 (Emsurg) *“Some days, I’d get out of bed, get downstairs, get on the settee and I’d be there all day until my missus came back from work. I just wouldn’t move”*.

## Discussion

This is the first study to explore transition from presurgery through to early post-operative recovery from the patients’ perspective following elective and emergency discectomy. The IPA approach offers new insight into symptoms experienced and effects on all aspects of life, gaining valuable reflections on the surgical journey including barriers, facilitators and perceived support requirements. Findings are concordant with positive outcomes from previous studies identifying physical improvements after discectomy, including reduced leg symptoms and disability,^3^ and also offers a wider and more holistic perspective.

The ‘ready to move forwards’ theme describes combined postoperative symptomatic and physical improvements with high satisfaction, positive emotions and optimism fuelling motivation towards achieving individual goals. These factors offer a strong platform for rehabilitation and recovery. However, the second theme describes concurrent ‘post-operative fear and uncertainty.’ Fear is an unpleasant emotion in response to perceived or recognised danger or threat; for participants, this is related to the threat of recurrent symptoms, return to difficult presurgery state and uncertainty for the future. These findings are similar to an Italian interview study^21^ which specifically investigated thoughts and concerns of 28 participants undergoing a range of lumbar surgical procedures (discectomy, decompression and arthrodesis fixation) at point of discharge. Our study focused on discectomy patients with interviews exploring a wider perspective of what was important to patients. However, similar outcomes included reports of unacceptable presurgery disability and lack of normal life as well as postoperative fear of pain recurrences. Our findings were also concordant with a previous interview study^15^ which described significant fear of pain generation and ‘undoing’ the effects of surgery with associated avoidant behaviours. It is recognised in the wider evidence base that pain-related fear and uncertainty can lead to safety-seeking behaviours potentially adversely affecting normal movement and activity, thus limiting progress with negative emotions and behaviours also associated with increased risk of chronicity and disability.^12^ Conversely, it has been shown that people who are not threatened by pain will follow recovery-orientated goals, facilitating faster recovery.^22^

Our third theme, ‘advice and guidance considered important’ describes the opportunity for healthcare providers to address such fears and uncertainties posing potential barriers to progress. Participants reported keenness for advice and guidance to help successfully navigate recovery and gain advice and reassurance when unexpected events and potential setbacks were experienced. These findings align with the Italian interview study,^21^ which included participants undergoing a range of lumbar operative procedures; although only verbal advice and guidance was provided, participants sought this perisurgical information, and support was valued. Furthermore, in concordance with a previous discectomy study,^15^ patient information resources were considered useful to enhance and reinforce verbal advice; strong compliance with provided advice and guidance was also reported. These findings highlight how Health Care Professionals (HCPs) are potentially powerful influencers on beliefs and behaviours postsurgery. It is therefore important that HCPs offer high-quality evidence-based perioperative advice, guidance and comprehensive patient information resources to positively influence recovery and challenge unhelpful beliefs and behaviours, thus empowering recovery. Within the non-surgical back pain literature, Darlow found that clinicians could provide reassurance which increased confidence with advice positively influencing the approach to movement and activity^23^. However, in Theme 3, participants reported incidences of repeated advice potentially fostering unhelpful beliefs and therefore behaviours, thus increasing the risk of developing persistent pain, maladaptive behaviours and limiting recovery. Similar findings were reported in a previous interview study^15^ focussing on physiotherapy post-operative experience; fear-avoidance behaviours were not challenged with resultant levels of post-operative anxiety described relating to movement and activity restrictions. It is beyond the scope of this study to explore if patient understanding diverged from the HCP intended message and highlights an area for future investigation.

In Theme 3, participants also described value in perioperative patient information resources, with instances of participants searching the internet for additional information and guidance when resources and advice provided did not fully meet their needs. However, Brooks^24^ analysed Lumbar Discectomy YouTube content against British Association of Spinal Surgeons (BASS) criteria with approximately 50% of content graded poor/inadequate quality. In another study,^25^ lumbar discectomy patient information available on NHS Trust websites was examined; 47% of identified leaflets contained poor/fair information, with 44% considered good quality and only 3% excellent. The limited evidence base underpinning patient information is a likely contributing factor to the confusing postoperative rehabilitation guidance for clinicians and patients. For example, a previous survey^26^ reported wide-ranging activity limitations postdiscectomy within UK practice with ongoing debate regarding postdiscectomy restrictions.^10^ Clearer evidence with international consensus guiding best peridiscectomy practice would facilitate more confident and positive influences on recovery.

While our results support advice and guidance for all, the final theme described ‘heterogeneity in peri-operative needs’. The first subtheme characterises variation in depth of and access to patient information. Two participants described reliance *only* on verbal guidance from preoperative consultations, but our findings indicate consequent limited understanding of expected progress reducing self-efficacy. There were also differences between emergency and elective surgery patient needs with the former ‘missing’ preoperative preparation. Therefore, ensuring access to comprehensive patient information, especially during and after emergency admission, is recommended, as well as encouragement for patient engagement with resources.

There was also variability in perceived postoperative support; while patient information with minimal postoperative HCP input was adequate for some, others wanted more detailed rehabilitation and guidance. Furthermore, findings demonstrated a spectrum of preoperative activity and functional levels (eg, ranging from asymptomatic and able to work until surgery to others describing prolonged presurgery very sedentary behaviours—see table 1 and Table of Participants online supplemental file). This heterogeneity has not been considered within clinical trials^5 9^ where discectomy patients have been treated as a homogenous group and potential benefits of post-op rehabilitation explored for *all* patients. It is therefore postulated that within previous studies, inclusion of those with higher preoperative functioning and postoperative symptom resolution may mask benefits for those who would benefit most. There were indications of transferable skills influencing self-efficacy (eg, one patient with previous history of depression described use of previously acquired cognitive behavioural therapy skills). However, it is beyond the scope of this study to identify subgroups requiring more detailed rehabilitation, but our findings support personalised perioperative management based on identified physical and psychosocial needs.

## Strengths and limitations

The use of IPA was a key strength of this study, providing detailed insight from participants. The main author was immersed in the study through data collection and subsequent analysis, although analysis was a collaborative process including researchers, clinicians and patient representatives; this triangulation was a strength. However, there may have been bias as all interviews were completed by the same individual; involving clinicians with different professional backgrounds may have reduced this potential bias. Effort was made to include a representative sample of discectomy patients including those undergoing emergency and elective procedures and wide-ranging backgrounds although none of our participants described dissatisfaction or specifically experienced perioperative complications. Participants were encouraged to freely describe their experiences providing depth of data. All participants underwent surgery within one secondary care NHS Trust, although external validity is a recognised limitation of IPA. The study indicates heterogeneity within patient rehabilitation needs, but it was beyond the scope of this study to identify criteria for those with low versus more intensive rehabilitation need, highlighting an area requiring future research.

## Conclusions

The findings highlight the transition from a difficult preoperative state to postsurgery improvement. There was postoperative high satisfaction, positivity and optimism for resuming previously enjoyed activities and improved quality of life. Clinicians have an opportunity to harness patients’ motivation towards achieving personalised goals and to address fears and uncertainties that could inhibit progress or even limit eventual outcomes; our findings demonstrate healthcare professionals’ powerful influence, with participants describing value in HCP input. However, results also suggest scope to improve quality of verbal communication to minimise iatrogenic negative healthcare messages as well as ensuring access to appropriate comprehensive information to support recovery. The final theme indicates that advice and guidance is adequate for some, empowering self-management. However, others require more intensive peri-operative input with results supporting personalised care based on individual biopsychosocial needs. This qualitative study identifies what is important from the patients’ perspective and areas to improve support for those undergoing discectomy towards improving the patient journey as well as optimising outcomes.

## Supplementary material

10.1136/bmjopen-2025-101259 online supplemental file 1

10.1136/bmjopen-2025-101259 online supplemental file 2

## Data Availability

Data are available upon reasonable request.
