# Supplementary material for: Patient lumbar discectomy journey (DiscJourn) in the UK: a qualitative study
Source: BMJ Open. 2025 Jul 25;15(7):e101259. doi: 10.1136/bmjopen-2025-101259 (PMC12306316; doi:10.1136/bmjopen-2025-101259)
Supplement: online supplemental file 1 [file bmjopen-15-7-s001.docx]

**Supplementary file Topic Guide; DiscJourn**

**Interview Schedule**

- Thank you for agreeing to participate in this interview
- This interview aims to understand your experiences related to your discectomy surgery both before, during and after
- It will take about 60-90 minutes to complete.

Before we start…..

**Consent**

You have already consented to take part in the interviews but I’m just checking you are still happy to participate.

- As we go through the interview, please let me know if you don’t understand any of the questions.
- No right or wrong answers – so please respond freely and honestly
- If there is a question you don’t want to answer we can move onto the next question
- Stop at any point without giving a reason
- Interview will be recorded but data will be kept securely and deleted after analysis
- Your answers and any quotes you make will remain anonymous and will not be reproduced in any way that would reveal your identity.
- Do you have any questions regarding the interview or how the data will be used?
- Do you give your consent to be recorded?

The interview will cover 3 main areas which are; your experiences before you had surgery; experience of surgery itself and post-operative experiences.

**Pre Operative Experiences**

**All of the following questions relate to the period of time (maybe months or years) before you had your surgery.**

1. Can you tell me first of all, a brief history of your back (leg) problems?
   - Description of symptoms before surgery

- Duration of problems
- Understanding of problem
- Previous interventions and results
- Most/ least helpful aspects of your management prior to surgery

1. What was your personal situation prior to surgery?

- Home life
- Family relationships
- Any issues in the family that were affecting you?
- Employment status
- Job description
- Hobbies/activiites

1. What impact did your condition have on your life prior to surgery?

- How did the back problem affect “your normal” activities/ personal situation?
- Did the condition affect your mental health in any way? (such as anxiety, depression/ low mood, ability to concentrate?)
- Did the back problem have any adverse impact on your friends or family?
- What prompted you to seek a referral from your GP to see the surgical team?
- Did you discuss the decision to seek a referral with your immediate family or friends? Did they influence the decision to seek a referral?

1. What (coping) strategies have you used prior to your surgery to help you manage or carry on despite your condition?

- Goal setting
- Pacing
- Family/friends
- Self belief
- Do you feel you were coping?
- Are there ways in which tou normally cope with things?

1. Why did you decide to go on the waiting list for surgery?

- Main factor
- Attitudes
- Beliefs
- Concerns or anxieties about surgery?
- Explore reasons for concerns/anxieties
- Did you do your own research?
- What did you find out?
- Did this make you think differently about the surgery?

1. What were your expectations/ goals with surgery? Who discussed these with you?

- Symptoms
- Family life
- Activities
- Work
- Were your expectations / goals discussed with anyone?
- Was it made clear to you what the surgery was designed to achieve?
- How confident did you feel that these goals would be achieved?
- Were you offered written information/ verbal information/ signposted to websites or patient information resources prior to surgery?

1. What were your expectations/ goals of the rehabilitation process following surgery?
   - What did you think it would involve?
   - Frequency?
   - Duration?
   - By whom
   - Was it discussed?
2. How supported did you feel during the period prior to your surgery?
   - By whom
   - Has it matched expectations?
   - More or less?
   - And at what point?
3. Prior to your surgery, can you sum up what your main positive and negative feelings were?
4. How did you prepare for your surgery?

- Yourself?
- Family?
- Friends?
- Emotionally/ practically?
- What support did you receive prior to admission and surgery? Is there anything that was not offered that you think would have been useful?/ (or offered that was not useful?)

**Experience of Surgery**

**Now please think about your recent surgery.**

1. How did you feel when you first woke up?
   - First thoughts?
2. Please tell me about your experience of the surgery itsefl.
   - Positive feelings
   - Negative feelings
   - Is it what you thought it would be like?/ match your expecations?
   - If not, why/ which aspects didn’t?
   - If yes, please explain
3. What is your understanding of the surgery that was carried out
   - Has it been discussed?
   - By whom?
   - When?
   - Is that what you thought was going to be carried out?
   - If no, what is different?
4. What is your understanding of your rehabilitation plan?

- Did you receive phsyiotherapy during your hospital stay?
- Did this match your expectations? (Was this useful/ in what way/ would you suggest any changes to the current physiotherapy provided during your hospital stay?
- Was the rehab programme (including after discharge home) discussed during your hospital stay?
- By whom?
- When?
- Was it what you expected? IS that what you thought was going to be the plan?
- If no, what was different?
- How do you feel about your rehabilitation plan?
- How confident do you feel that you will adhere/ achieve your rehabilitation plan?

1. How supported did you feel during your surgery/time in hospital?

- By whom?
- Has it matched your expecations?
- More or less?
- And at what point?

1. At the time of your surgery, what was your personal situation?

- Home life?
- Family relationships?
- Any issues in the family that were affecting you?
- Employment status?

**Post Operarative Experiences**

**All of the following questions relate to the period of time since you were discharged from hospital following your surgery.**

1. How do you feel that you are recovering at the moment?

- Does it match expectations?
- If not, why not?
- If yes, please explain
- Facilitators to recovery?
- Barriers to recovery?
- Awareness of advice to enable recovery?

1. Do you feel able to follow / adhere to advice and guideines offered to you during the pre operative period and during your time in hospital?
   - If yes please explain

- If not, please explain
- Facilitators to encourage adherance?
- Barriers to adherance?

1. What were your expectations / goals of the rehabilitation process following surgery?

- What did you think it would involve?
- Frequency
- Duration
- By whom
- Was it discussed?
- Carer support?

1. What are your plans with regards to returning to normal Activities of Daily living?

- Functional activities?
- Wider activites relevant to you?
- Work?
- Facilitators to the above?
- Barriers to the above?
- How did you make these plans?
- If you havent made plans, why not?

1. What (coping) strategies have you used since your surgery to help you manage or carry on?

- Goal setting
- Pacing
- Family/ friends
- Self belief

1. How supported have you felt since your discharge from hospital following your surgery?

- More or less? Please explain
- By whom
- Has it matched your expectations?
- And at what point?

1. What is your personal situation at the moment?
   - Home life
   - Family relationships
   - Any issues in the family that are affecting you?
   - Employment status
   - Job description
   - Hobbies/activiites
2. During this early stage of your rehabilitation, can you sum up your what your main positve and negative feelings are?

**Thinking about the future…**

What are your key thoughts about the recovery ahead of you?

Thank you for all your comments. Is there anything else you would like to add before we end the interview?

That concludes the interview.

Thank you for your time – your help is very much appreciated.

The interview will now be transcribed.

Through a process called member checking, I would like to offer you the opportunity

to read through your transcript and add any further reflections. This process can be

completed through post or email with the opportunity to discuss by telephone/ skype.

At this stage, what are your thoughts on how would you like to receive the transcript?

Check- how participant is getting on with the weekly diary?

Any issues- or things I can help with?

Do you need any more copies?
