# Supplementary material for: Patient lumbar discectomy journey (DiscJourn) in the UK: a qualitative study
Source: BMJ Open. 2025 Jul 25;15(7):e101259. doi: 10.1136/bmjopen-2025-101259 (PMC12306316; doi:10.1136/bmjopen-2025-101259)
Supplement: online supplemental file 2 [file bmjopen-15-7-s002.docx]

| **Participant number** | **Elective or Emergency** | **Symptom duration** | **Employment status** | **Pre-op Activity level** |
| --- | --- | --- | --- | --- |
| 1 | Elective | 18 months | Employed- working | Gardening, actively exercising |
| 2 | Elective | 9 months | Not working | Sedentary pre-op due to pain |
| 3 | Emergency | 10 months | Self employed- Off work | Sedentary due to pain |
| 4 | Elective | 18 months | Retired | Active- gardening and DIY |
| 5 | Emergency | 7 yrs back pain; 4 yrs leg pain; acute cauda equina syndrome | Employed - working till day of admission | Active; walking trying to exercise. |
| 6 | Elective | 12 months back pain only; 12 months back and leg symptoms. | Employed- working | Trying to stay active e.g. walking dog, family activities but limited |
| 7 | Elective | 18 months | Employed- working till week before surgery but limited | At work but supported by colleagues; avoided manual duties e.g. mopping, hoovering; didn’t go out much apart from to work. |
| 8 | Elective | 12 months | Self employed- Minimal working pre-surgery | Trying to stay active; previously very fit and keen runner/ gym |
| 9 | Emergency | 3.5 years of back pain with intermittent bilateral leg pain, numbness and foot paraesthesia | Employed- working till day before surgery | Tried to remain active but had stopped martial arts; limited exercise at gym |
| 10 | Elective | 11 months | Employed- office based accounts manager | Working till day before surgery; had 3 months off work since onset of symptoms |
| 11 | Excluded |  |  |  |
| 12 | Emergency | 1 week | Employed- nurse. Working day admitted | No pre-surgery functional restrictions- was working day before surgery |
| 13 | Elective | 15months | Retired | sedentary due to pain |
| 14 | Excluded |  |  |  |
| 15 | Emergency | 1 week | Employed- At work till day before surgery. | Active without restriction until week before surgery |
| 16 | Emergency NB- was already on the waiting list for elective surgery. | 2years back pain; gradually worsening leg pain;acute motor weakness pre-surgery. | Employed- Office based work for cancer Charity | Limited e.g. difficulty walking, shopping, driving, family activities |
