# Supplementary material for: Patient lumbar discectomy journey (DiscJourn) in the UK: a qualitative study
Source: BMJ Open. 2025 Jul 25;15(7):e101259. doi: 10.1136/bmjopen-2025-101259 (PMC12306316; doi:10.1136/bmjopen-2025-101259)
Supplement: Supplementary file 3 [file bmjopen-2025-101259.reviewer_comments.pdf]

PEER REVIEW HISTORY

BMJ Open publishes all reviews undertaken for accepted manuscripts. Reviewers are asked to complete a checklist review form and are provided with free text boxes to elaborate on their assessment. These free text comments are reproduced below.

ARTICLE DETAILS

Title (Provisional)

The patient lumbar discectomy journey (DiscJourn) in the UK: A qualitative study

Authors

White, Louise; Heneghan, Nicola R; Furtado, Navin; Baraks, Karl; Parvez, Zeeshan; Masson, Annabel; Rushton, Alison B

VERSION 1 - REVIEW

**Reviewer**	**1**
**Name**	**Ferrari, Silvano**
**Affiliation**	**Universita degli Studi di Padova, Molecular Medicine**
**Date**	**20-Mar-2025**
**COI**	**None**

The patient lumbar discectomy journey (DiscJourn): A qualitative study

This article is very interesting and well conducted and will certainly help the scientific community to improve the knowledge of the patients' experiences during the spinal surgery.

I would just like to make a few notes to the authors:

1) The type of symptoms which patients have complained is deduced only from the table of participants. From this table it would seem that the pre-surgery symptomatology was heterogeneous (subjects with radiculopathy and subjects without radiculopathy). I believe that this situation should be highlighted in the abstract and in the text.

2) METHODS

1. Design - A Study Management Group (SMG) met regularly to oversee study quality. How many were there and which professional figures were made up from SGM?

a. Partecipants – Potentially eligible participants were identified (n=22) by neurosurgery team members including hospital Principal Investigator (PI) (LW), surgeons, ward physiotherapists and waiting list coordinators prior to or during admission. Do the "neurosurgery team members" correspond to the SMG?

1. Participant Demographics - Three subjects had Co-existing past medical history (2 depression and one panic attacks). Why did the authors decide to keep them included? How were the answers of these patients inside the interview? Wouldn't it be interesting to check if they are consistent with expectations and possibly include them in the discussion?

b. Data collection - Semi-structured interviews were undertaken by the PI (LW), a specialist physiotherapist, between May 2018 and March 2019. Who prepared the semi-structured interviews?

c. Data analysis - Stage 2 - In accordance with IPA, emergent themes were identified (LW) and presented to the SMG (including patient co-investigator) for examination and discussion. In this step it would seem that the topics were identified by a single interviewer but in the next step (stages 3 and 4) it seems that this identification was performed by two people (The PI and blind reviewer (ZP) Can you clarify these passages? In addition: what professional figure was the blind reviewer?

1. In the procedures described, there is no mention of "triangulation" as research strategies to improve the accuracy of the work through the convergence of information and data processing from different sources. Why is it not mentioned? It may have been done somehow, but it should be described better.

DISCUSSION

a. The Autors stated that “This is the first study to explore transition from pre-surgery through to early post-operative recovery from the patients’ perspective following elective and emergency discectomy.” I think it is partially true. I suggest to read this paper S. Ferrari, C. Cedraschi, N. Mapelli, A. Baram, F. Costa, R. Gatti & M. Fornari(2022): Thoughts and concerns of patients at hospital discharge after lumbar spine surgery. A qualitative study. Disability and Rehabilitation, DOI: 10.1080/09638288.2022.2148761

1. Insight the discussion, about the theme 2, “Similar findings were reported in a previous interview study[20] focussing on physiotherapy post-operative experience”. I believe that the bibliographic reference in this phrase (20) is incorrect.

**Reviewer**	**2**
**Name**	**Greenwood, James**
**Affiliation**	**National Hospital for Neurology and Neurosurgery**
**Date**	**30-Apr-2025**
**COI**	**None**

Dear authors many thanks for affording me the opportunity to review this manuscript.

You have delivered a good paper evaluating the patients experiences of lumbar disc surgery. It is well written and adds value to the existing knowledge base.

I wondered if the was a small typo at the end of the PPI section, consents forms, apologies if consents was deliberate.

To my mind the introduction nicely establishes the varied outcome post discectomy but the sample seemed not to include those with a poor surgical outcome. You discuss this limitation and make suggestions for future studies which are proportionate.

The conclusions regarding individualised care seem supported buy this and others work.

I very much enjoyed reading this.

I wish you and your team all the very best of luck with your research endeavours.

VERSION 1 - AUTHOR RESPONSE

**Reviewer 1**	Thank you for your feedback. Responses to each point is outlined below.
The type of symptoms which patients have complained is deduced only from the table of participants. From this table it would seem that the pre-surgery symptomatology was heterogeneous (subjects with radiculopathy and subjects without radiculopathy). I believe that this situation should be highlighted in the abstract and in the text.	The supplementary table of participants has been refined to ensure anonymity (in line with the editors recommendations). Participant demographics are also summarised in Table 1 (page 9-10). It can be confirmed that all participants experienced pre-surgery radiculopathy and therefore no change has been made in the text or abstract.
METHODS<br>1. Design - A Study Management Group (SMG) met regularly to oversee study quality. How many were there and which professional figures were made up from SGM?	Methods (page 6)- “Design” paragraph.<br>The Study Management Group comprised of 6 individuals; a PPI representative, 2 clinicians (physiotherapists), 2 academic colleagues, and a post-graduate physiotherapy student. Detail has been added to explain this in the text.
a. Participants – Potentially eligible participants were identified (n=22) by neurosurgery team members including hospital Principal Investigator (PI) (LW), surgeons, ward physiotherapists and waiting list coordinators prior to or during admission. Do the “neurosurgery team members” correspond to the SMG?	Participants (Page 6)<br>Only the PI (LW) was included in the neurosurgery team and SMG. No changes have been made in the text.
1. Participant Demographics - Three subjects had Co-existing past medical history (2 depression and one panic attacks). Why did the authors decide to keep them	Patient demographics (in “Participants” paragraph, page 6 and Table 1- page 9-10)

included? How were the answers of these patients inside the interview? Wouldn't it be interesting to check if they are consistent with expectations and possibly include them in the discussion?	It was considered that patients seen within normal clinical practice often report past medical problems and so participants who reported mental health history were not excluded from the study. Also, neither of the patients with history of depression were in a depressive state at the time of undergoing surgery/ interview and all were able to participate in the interviews to the same extent as others.<br>It was beyond the scope of this study to specifically explore if such histories affected outcomes or to identify subgroups of patients although there was evidence of transferable skills (table 5, subtheme "Heterogeneity in perceived post-operative support", P15) which related to use of previously acquired skills for managing depression.<br>An additional line has been added to the discussion relating to this finding – final discussion paragraph page 20. This is certainly an area of interest for future research.
b. Data collection - Semi-structured interviews were undertaken by the PI (LW), a specialist physiotherapist, between May 2018 and March 2019. Who prepared the semi-structured interviews?	Data collection (page 6)<br>There was no funding to support administration within the study and therefore PI (LW) prepared/ arranged the interviews. This has been made clearer within the text with the study protocol publication included in references (Ref 18).
c. Data analysis - Stage 2 - In accordance with IPA, emergent themes were identified (LW) and presented to the SMG (including patient co-investigator) for examination and discussion. In this step it would seem that the topics were identified by a single interviewer but in the next step (stages 3 and 4) it seems that this identification was performed by two people (The PI and blind reviewer (ZP) Can you clarify these passages? In addition: what professional figure was the blind reviewer?	Data analysis (pages7-8)<br>Thank you for drawing attention to this. The text has been amended in this section to clarify the process with initial analysis undertaken by LW and ZP and more detail has been added.<br>The blind reviewer was a post-graduate Physiotherapy student; this detail has been added in an earlier section (first paragraph, page 6 - SMG membership details have been added).
1. In the procedures described, there is no mention of "triangulation" as research strategies to improve the accuracy of the work through the convergence of information and data processing from different sources. Why is it not mentioned? It may have been done	Procedures- In "Data analysis" pages 7-8.<br>Various strategies were included to improve accuracy of this work which are mainly included in "Trustworthiness" section (page 8). Further details have been added to

somehow, but it should be described better.	highlight how data processing involved independent analysis from 2 researchers and then regularly with the SMG.<br><br>In strengths and limitations, (point 2, page 3 and in paragraph on page 20) the collaborative analysis process (including triangulation) has been explained in more detail to enhance appreciation of accuracy of work.
DISCUSSION<br>a. The Authors stated that “This is the first study to explore transition from pre-surgery through to early post-operative recovery from the patients’ perspective following elective and emergency discectomy.” I think it is partially true. I suggest to read this paper S. Ferrari, C. Cedraschi, N. Mapelli, A. Baram, F. Costa, R. Gatti & M. Fornari(2022): Thoughts and concerns of patients at hospital discharge after lumbar spine surgery. A qualitative study. Disability and Rehabilitation, DOI: 10.1080/09638288.2022.2148761	Discussion<br>Thank you for alerting me to this reference. This is a very interesting study. The conclusions align well with our study findings and have been included within the discussion section (Page 17 and 18). Similarities and differences have been integrated; for example, we focussed solely on patients undergoing primary discectomy surgery whereas Ferrari et al included patients undergoing a wider range of surgical procedures for degenerative diseases. Also, Ferrari’s interviews were at the point of discharge whereas our interviews were later (post-discharge) with content analysis employed rather than analysis in line with IPA in our study. In addition, there were differences in the aims of these studies; Ferrari et al aimed to investigate thoughts and concerns of patients undergoing surgery for degenerative disease at the time of discharge whereas our study took a wider view of the patient perspective and lived experiences of primary discectomy surgery.<br><br>Final reference numbers have been amended in line with adding this additional publication to the reference list.
1. Insight the discussion, about the theme 2, “Similar findings were reported in a previous interview study[20] focussing on physiotherapy post-operative experience”. I believe that the bibliographic reference in this phrase (20) is incorrect.	Reference 20 is incorrect (page 18) - thank you for drawing this to our attention. The reference should be number 15 and has been amended.

Reviewer 2<br><br>I wondered if the was a small typo at the end of the PPI section, consents forms, apologies if consents was deliberate.	Thank you for your comments.<br><br>You correctly noticed a small typo a the end of the PPI section on page 8. The “s” has been removed at the end of “consent forms”.

VERSION 2 - REVIEW

Reviewer **1**

Name **Ferrari, Silvano**

Affiliation **Universita degli Studi di Padova, Molecular Medicine**

Date **04-Jul-2025**

COI

I have no other remarks to make.

Compliments
